# Muecas: A Multi-Sensor Robotic Head for Affective Human Robot Interaction and Imitation

**DOI:** 10.3390/s140507711

**Published:** 2014-04-28

**Authors:** Felipe Cid, Jose Moreno, Pablo Bustos, Pedro Núñez

**Affiliations:** RoboLab, Robotics and Artificial Vision Laboratory, University of Extremadura, Escuela Politécnica, Avenida de la Universidad s/n, Cáceres, Spain; E-Mails: josemore@unex.es (J.M.); pbustos@unex.es (P.B.); pnuntru@unex.es (P.N.)

**Keywords:** human robot interaction, robotic head, imitation

## Abstract

This paper presents a multi-sensor humanoid robotic head for human robot interaction. The design of the robotic head, Muecas, is based on ongoing research on the mechanisms of perception and imitation of human expressions and emotions. These mechanisms allow direct interaction between the robot and its human companion through the different natural language modalities: speech, body language and facial expressions. The robotic head has 12 degrees of freedom, in a human-like configuration, including eyes, eyebrows, mouth and neck, and has been designed and built entirely by IADeX (Engineering, Automation and Design of Extremadura) and RoboLab. A detailed description of its kinematics is provided along with the design of the most complex controllers. Muecas can be directly controlled by FACS (Facial Action Coding System), the *de facto* standard for facial expression recognition and synthesis. This feature facilitates its use by third party platforms and encourages the development of imitation and of goal-based systems. Imitation systems learn from the user, while goal-based ones use planning techniques to drive the user towards a final desired state. To show the flexibility and reliability of the robotic head, the paper presents a software architecture that is able to detect, recognize, classify and generate facial expressions in real time using FACS. This system has been implemented using the robotics framework, RoboComp, which provides hardware-independent access to the sensors in the head. Finally, the paper presents experimental results showing the real-time functioning of the whole system, including recognition and imitation of human facial expressions.

## 1. Introduction

The creation and design of social robots has drawn attention to many fields of science. Most of the current robotic platforms are focused on improving the existing methods for human-robot interaction (HRI), providing the robot with affective skills and behaviors. Social robots follow behaviors similar to humans: they interact and communicate with humans by following a set of social rules, e.g., by using modalities of communication also used in human-human interaction (such as speech, facial expressions or body language). Interesting studies that combine psychological and robotics theories have demonstrated that the mechanical design of the platform and the social skills with which the robot is programmed would increase the empathy and the acceptance level of the robot [1,2]. In this respect, the emotional states of the user and how the robot is able to estimate them are one of the most critical processes in natural human-robot interaction (HRI). Thus, knowing and understanding these human emotions helps social robots to adapt their communication in real time, improving and enriching the interaction [3]. This kind of HRI is usually known as affective HRI, which has become a central part of the social robotics field in recent years.

In affective scenarios, the design of robots that take into consideration not only the position and the information of the user's environment, but also emotional information, is desirable. On the one hand, the estimation of the emotional state of the human has a high degree of complexity in HRI. Most of the affective HRI techniques use only one information channel, called the mode, in order to recognize human emotions. In this respect, the facial expressiveness is usually one of the richest and most powerful sources of affective information. However, several papers work with more than one input mode, and it is common to find multimodal emotion recognition systems in the literature. The robustness and accuracy of these latter systems are improved in this kind of affective scenario [4,5]. Speech, body language or a skeletal modeling [6,7] are commonly used as inputs to the system. On the other hand, a robot able to imitate emotions and basic movements also presents several advantages in affective HRI. First, the robotic agent increases its acceptance level and empathy. Besides, the robot is able to express emotions and modify the user's emotional state [8].

There are two main contributions of this paper. First, a new design for a multi-sensor humanoid robotic head, called Muecas, is proposed. The platform is equipped with a wide set of sensors and actuators that allows recognizing and interacting with humans in real scenarios. The robotic head has a friendly appearance for affective HRI and presents a perception system composed of a set of sensors to acquire information from the world and the user in a similar way to humans. The multi-sensor robotic head is equipped with a stereo vision system, an RGBD camera, an inertial sensor and an audio system. Muecas is also designed to allow a wide set of expressive movements, most of them associated with natural language. Thus, Muecas is able to generate pitch, yaw and roll movements on the neck, in conjunction with other movements more related to basic facial expressions (e.g., eye pan, eye tilt and eyebrows, among others). Secondly, this paper also presents a human emotion recognition and imitation system. On the one hand, the recognition of human emotions is performed using a multimodal approach. The proposal uses a Bayesian solution in order to detect the human emotion from both the facial expressiveness and human speech. The use of classifiers, such as the Bayesian approach, represents a common solution in the literature for emotion recognition systems from facial expressions. However, there are many alternatives to this type of classifier, such as support vector machine (SVM) [9], model tree [10], binary decision tree [11] and neural networks [12], among others. Dynamic Bayesian networks are an interesting solution for emotion recognition from dynamic images. They use a set of consecutive frames to estimate the human emotion, and the properties of the network are dynamically updated, ensuring that it converges, as is demonstrated in the Experimental Results. On the other hand, the imitation system allows the mapping of the emotion detected by the robot into a set of mechanical movements. A model-based representation is used in order to avoid physical collisions and to anticipate the movements or results of the robot reaction.

This paper is organized as follows: after discussing previous works in the literature related to the design of robotic heads and the state-of-the-art in human emotion recognition and imitation systems, Sections 2 and 3 present a detailed description of the hardware and software architecture of the multi-sensor humanoid robotic head, Muecas. In Section 4, an overview of the proposed multimodal recognition and imitation system is presented. Next, Section 5 outlines the experimental results, and finally, Section 6 describes the conclusions and future work for the presented approach.

## 2. Related Works

To achieve natural human-robot interaction, the design of social platforms and how this design affects the interaction has been inspired for a long time by physiological and biological studies [13]. Most of the research has shown the importance of giving the robot a human-like appearance [14]. In the current robotic literature, these robots are composed of hardware and software systems that allow performing actions or expressing emotions in a similar way to humans. Usually, these robots are designed with natural language interaction capabilities. Natural language is composed of both verbal and non-verbal communication; thus, the state-of-the-art in this topic for HRI is very broad. From human emotion recognition and imitation to the understanding of body language or speech recognition and generation, an extensive list of research and works is described in the literature [15–18].

Specifically, several works in the design of robotic heads for natural HRI are based on the analysis and the imitation of human facial expressions. Most of the approaches are based on Paul Ekman's work [19,20]. This author identifies and classifies facial expressions through the study of different facial muscles in the human face (action units, AU), giving rise to the so-called Facial Action Coding System (FACS). The use of AUs establishes a homogeneity between different recognition and imitation systems of facial expressions and movements, allowing different imitation systems to be compatible with multiple robotic heads (the reader can refer to some interesting surveys in [21]).

In the field of affective HRI, the development of robotic heads capable of generating facial expressions has come to improve the empathy and the interaction with the user [2,22]. Some robotic heads have a limited number of facial expressions by using flashing LEDs [23,24]. Other works, in contrast, have a lot of degrees of freedom, using a set of mobile elements to generate and imitate the movements and the facial expressions (e.g., robotic mouth, eyes, neck or eyebrows). Some examples of these robotic heads are the robots, Kismet [25], ROMAN [26,27], KHH [28], WE-4RII [29] or SAYA [30,31]. Similar to these robots, Muecas is equipped with a set of mechanical elements that allows the performing of a broad range of natural movements and emotions.

Finally, the robot's capability of imitating facial expressions and human movements is a key factor that determines the design requirements of the heads used in social robotics. Usually, the imitation of facial expressions is achieved through mobile elements of the robotic head (e.g., eyelids, eyebrows, eyes or mouth). In this regard, four different categories can be found in the literature: anthropomorphic, zoomorphic, caricatured and functional. The robotic heads, BARTHOC [32], SAYA [30] or iCub [23] present an anthropomorphic appearance, which has many advantages for intuitive usage and understanding. Other studies have demonstrated that the use of robots with zoomorphic design helps to avoid the uncanny valley [33], establishing human-creature relationships. The robots, iCat [34] and Probo [35], are interesting examples of zoomorphic designs. Caricatured and functional designs are also commonly used in the literature for building robots. MIT's robotics head, Kismet, is an example of a caricatured robotic creature with perceptual and motor modalities for natural HRI. Table 1 compares the main features of different robotic heads in relation to the DoF of the robotic head, the sensors and the use or not of action units for emotion recognition and/or imitation [36].

## 3. Multi-Sensor Robotic Head for Human Robot Interaction

In this section, the design of the multi-sensor robotic head, Muecas, is presented. Muecas has been designed to study and compare new methods for natural HRI that help to improve attention and empathy during the communication. The main key is a simple design that takes into consideration several studies on social robot design and the importance of the appearance in the interaction. Different concepts, such as the shape, features, level of rejection of the design and its compatibility with natural language, has been taken into account.

Muecas has 12 degrees of freedom that are distributed as follows: neck (four), mouth (one), eyes (three) and eyebrow (four). The design requirements of the robotic head, Muecas, aims to combine these mobiles elements and sensors to establish an affective HRI through a human-like appearance and the use of facial expressions. Thus, a perception system is integrated in the architecture, which consists of a set of sensors and software modules. On one hand, Muecas is equipped with an audio system (*i.e.*, microphones and speaker), inertial system (*i.e.*, compass, gyroscope and accelerometer) and video and depth acquisition systems (*i.e.*, stereo cameras and RGBD sensor). On the other hand, the software modules consist of different subsystems for the human emotion recognition and imitation.

In the next subsections, a detailed description of the hardware architecture, as well as the software modules of the multi-sensor robotic head, Muecas, for natural human-robot interaction is described.

### 3.1. Design Requirements

This subsection introduces the design requirements of the multi-sensor robotic head described in this paper. Muecas has to be equipped with different sensors in order to acquire information of the robot's surrounding. Besides, the robotic head has to be designed to express emotions and to imitate the natural language of humans. The final design has taken into account not only the functions or abilities to understand and respond to the context of the communication, but also other concepts, such as the appearance or the acceptance level of the robot.

A biologically inspired approach has been adopted for the design and manufacturing of the proposed robotic head. Similar to other works, Muecas uses different systems that are based on psychology, cognitive architectures or the structure of interaction, among others [21]. On the one hand, as the morphology of the robotic head changes the perception and the response of the user in the interaction, it is presented in the proposed design as a significant factor. On the other hand, the level of realism is also a decisive factor that determines the emotional response of the user (*i.e.*, a highly realistic appearance presents an obvious rejection response, as is described in the uncanny valley theory [33,37]).

Zoomorphic designs allow one to create a simple relationship between users and robots (*i.e.*, master-slave), avoiding the aforementioned uncanny valley. These designs have unrealistic features and are considered in many cases as a toy by the user. On the contrary, anthropomorphic designs have forms and characteristics similar to humans, and they are commonly used in social robots that attempt to imitate a human-human interaction. Even so, an anthropomorphic approach generally presents problems with the uncanny valley when trying to build a robotic platform with high realism, which, consequently, focuses the attention of the user mainly in the differences and not in the common features. Finally, unlike anthropomorphic and zoomorphic designs, caricatured approaches use hardware pieces that are clearly disproportionate in size or shape, which represent the elements of the user's face. These designs are unrealistic; however, they easily attract the attention of the user. These latter approaches avoid the uncanny valley, resulting in a positive emotional response from the user compared to highly realistic anthropomorphic or zoomorphic designs.

**Figure 1.**
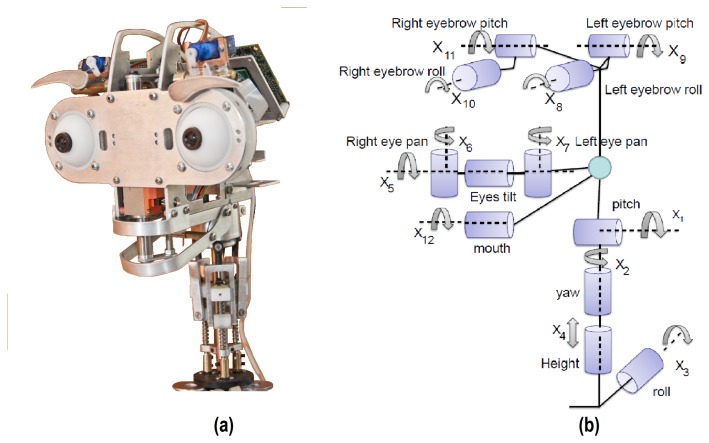
**(a)** The robotic head, Muecas, described in this paper; and (b) the kinematic chain of the multi-sensor robotic head, where *X_i_* represents each mobile element.

In this respect, a caricatured design has been chosen as a solution for the multi-sensor robotic head, Muecas. The proposal has a set of hardware components that improve the empathy and that attract the attention of the user (e.g., eyes, eyebrows or the mouth). Besides, the kinematic structure of the human body has a high degree of complexity, mainly in the movements of the neck, mouth or eyes. This complexity is an obstacle in current robotic platforms that attempt to imitate the natural language of the users, whereby, even when the platform implements an equal number of degrees of freedom for each element of the head, the movement is often different or limited in relation to that performed by the user. Therefore, the proposed approach does not attempt to imitate the anatomy and physiology of human head elements, but rather obtains a natural movement based on the human behavior that allows the user to identify and understand the body language and the facial expressions of the robot. Figure 1a illustrates the set of mobile components of the robotic head. In Figure 1b, the kinematic chain of the multi-sensor robotic head is shown, where *X_i_* represents each mobile element. These elements are described next.

#### 3.1.1. Neck Mechanism

**Figure 2.**
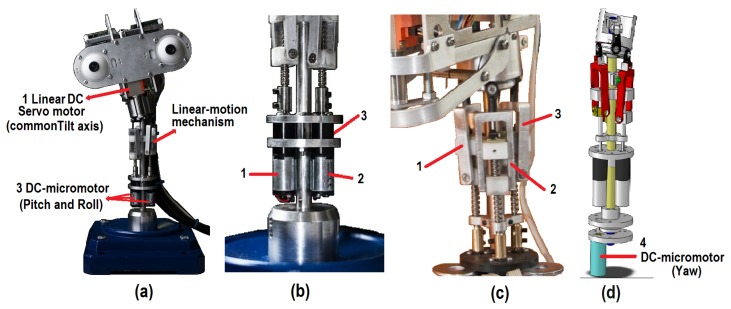
**(a)** Neck mechanism of the robotic head; (b) configuration of the motors associated with the pitch and roll movements; (c) configuration of the linear-motion mechanisms; and (d) DC-micromotor associated with the yaw movement.

The neck in a robotic platform is a key element in the imitation of human body language. As shown in Figure 1b, the proposed neck mechanism has four DoFs (*i.e., X*_1_, …, *X*_4_), which are associated with the pitch, yaw, roll and elevation movements, respectively. In more detail, the final configuration of the mechanical structure is drawn in Figure 2. The design of the neck has a base for a spherical joint shaft, three DC-micromotor 1724-024 SR (Encoder IE2-16 with 76:1 gear ratio) and a structure of movement based on linear-motion mechanisms (Figure 2a). This configuration is mentioned in several studies as parallel neck [23], although it presents several differences from the proposed design.

First, to generate the DoF associated with the pitch and roll movements, the positions of the three linear-motion mechanisms in relation to the type of movement are combined and synchronized (as shown in Figure 2b,c, respectively). The pitch movement uses two linear-motion mechanisms (1 and 2) in front of the neck, in the opposite direction to the linear-motion mechanism (3) (*i.e.*, in the back of the neck). In the case of the roll movement, one of the two linear-motion mechanisms in front of the neck (1 or 2) is used, in the opposite direction of the other linear-motion mechanism (2 or 1, respectively). Besides, the position of the linear-motion mechanism (3) on the back of the neck is decreased in relation to the roll angle.

Next, the yaw movement is obtained by means of a DC-micromotor (4) at the bottom of the structure and connected to the spherical joint shaft, which allows the rotation of the DC-micromotor and the upper section of the head (Figure 2d).

Finally, three DC-micromotors (1, 2 and 3), in conjunction with the linear-motion mechanisms, are used to generate the elevation movement. This mechanism is synchronized to raise and lower the height of the head.

#### 3.1.2. Eye Mechanism

**Figure 3.**
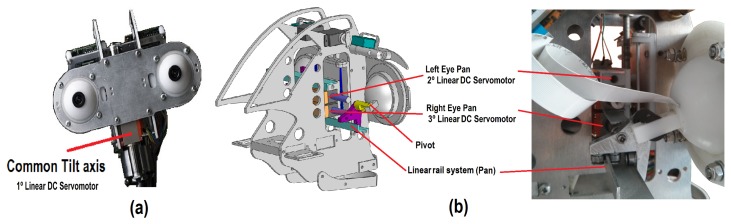
Configuration of the eyes mechanism: (a) common tilt axis; and (b) pan axis for each eye.

The proposed design is intended to imitate the natural movement of human eyes. Therefore, a common tilt for both eyes and an independent pan for each ocular globe has been used (*i.e.*, three DoF labeled as *X*_5_, *X*_6_ and *X*_7_ in Figure 1b). Figure 3a shows the tilt movement, which uses a Faulhaber LM-2070 linear DC servomotor (1). This servo motor vertically displaces the linear rail system joining the pivots that control the pan movement of the eyes (Figure 3b).

The pan movement is independent for each ocular globe and uses two Faulhaber LM-1247 linear DC-servomotors (2 and 3 for the left and right eye pan movements, respectively).

#### 3.1.3. Eyebrow Mechanism

**Figure 4.**
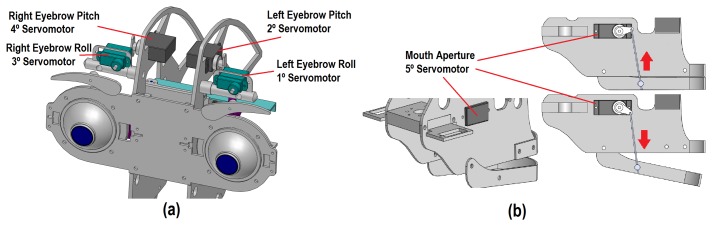
Configuration of the eyebrows and the mouth mechanism.

The use of eyebrows in imitation systems allows one to generate realistic facial expressions with different degrees of intensity. The proposed mechanism has four DoF (left eyebrow roll and pitch movements and right eyebrow roll and pitch movements, labeled as *X*_8_ to *X*_11_ in Figure 1b, respectively). The eyebrows mechanism has a different design in relation to other mobile elements of the robotic head, because each movement performed by an eyebrow is independent of the other (see Figure 4). Four HITEC HS-45HB servomotors have been used in the design.

First, the pitch movement is generated by the rotation of a servomotor (2 or 4 in Figure 4a), which changes the position of a metal support. This support exercises the turning force of the previous servomotor to rotate the base of the second servomotor connected to the eyebrow (1 or 3, respectively).

Finally, each eyebrow is connected directly to the servomotor (1 and 3) to generate for the roll movement. Therefore, an eyebrow can rotate in the same direction of rotation as the motor, as Figure 4a shows.

#### 3.1.4. Mouth Mechanism

The mouth of the robot has only one degree of freedom to imitate the movement of the human mouth (*i.e.*, the aperture of the mouth, which is labeled as *X*_12_ in Figure 4b). The aperture of the mouth is performed using the HITEC HS-45HB servomotor (5) and a metallic support. This support changes the position of the jaw of the robotic head in relation to the rotation angle of the motor, as illustrated in Figure 4b.

### 3.2. Hardware Architecture

**Figure 5.**
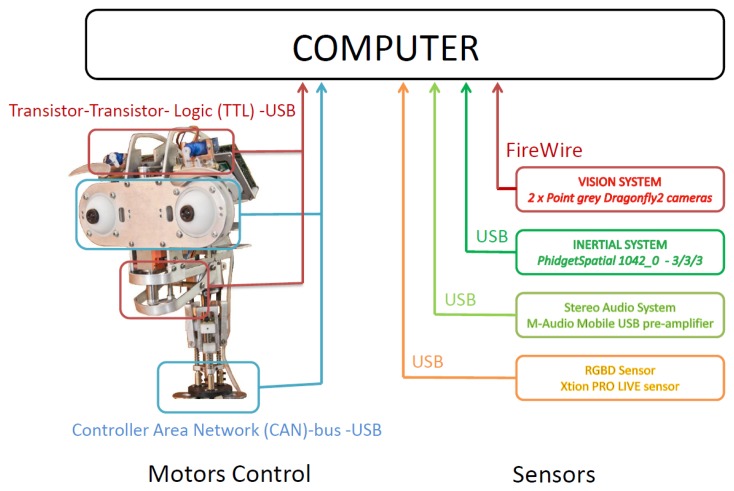
Hardware of the robotic head, Muecas.

An overview of the hardware used in the multi-sensor robotic head presented in this paper is outlined in Figure 5. Muecas uses a set of sensors and processing systems in order to acquire information of the surroundings and of the users in the interaction. Besides, a set of mobile elements are combined in order to generate facial expressions and to interact with humans (*i.e.*, actuators). The motors control system and the sensors with which Muecas is equipped are described in the following subsections.

#### 3.2.1. Motor Control Systems

**Figure 6.**
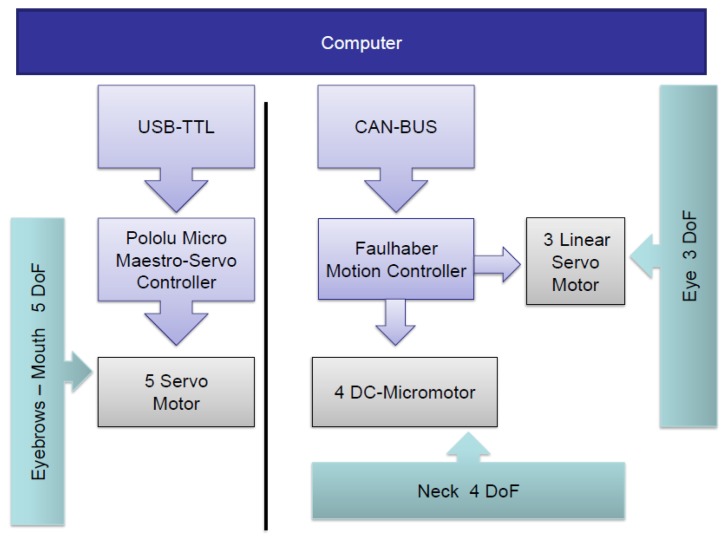
Architecture of the motor controllers.

The architecture of the two control systems is shown in Figure 6. On the one hand, the five servomotors associated with the eyebrows and the mouth are managed by the Pololu Micro Maestro 6-Channel USB Servo Controller. A USB to Transistor-Transistor Logic (TTL) serial cable is used for the communication between the controller and the computer. The four DC-micromotors and the three linear servomotors associated with the neck and eyes, respectively, are managed by the MCLM-3003 and MCDC-3003 Faulhaber motion controllers. The communication between these motor controllers and the computer is performed by a USB to Controller Area Network (CAN) adapter and the CAN bus protocol [38].

#### 3.2.2. Sensors

The current technology in the development of robotic heads allows the robot to perceive the world in a similar way to humans. In this respect, the robot is equipped with a set of sensors and systems that intend to mimic human senses. Next, the specific systems with which the robot is equipped are described in detail.

**Figure 7.**
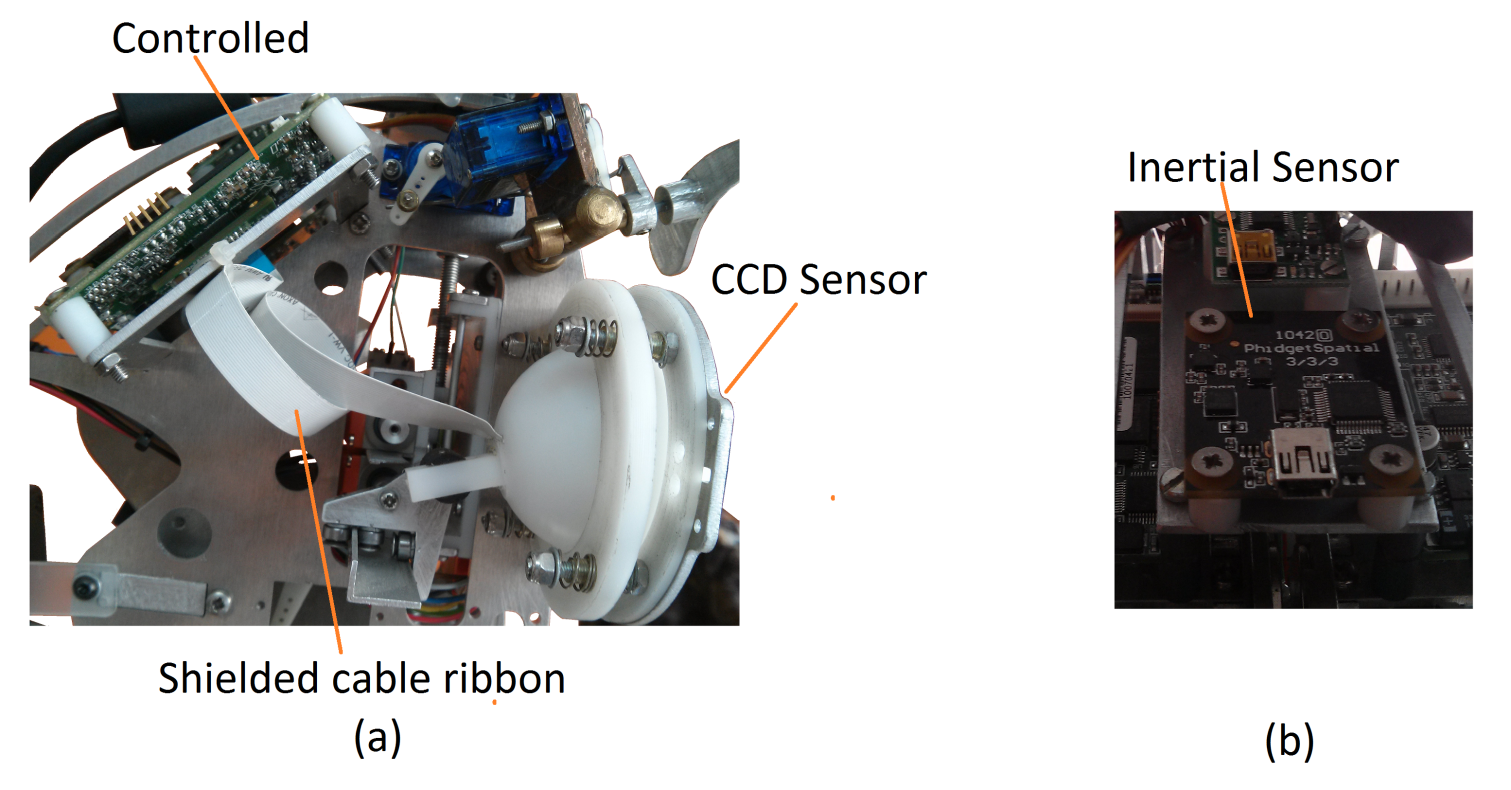
**(a)** Stereo vision system of Muecas; and (b) inertial system.

- Stereo vision system: Muecas is equipped with a stereo vision system based on two Point Grey Dragonfly2 IEEE-1394 cameras (DR2-13S2C/M-C). In each ocular globe, this system integrates a CCD sensor with a controller located on the lateral side of the robotic head, with an extended version that allows the CCD sensor to be located at a maximum distance of 15 cm of the controller of the camera with a shielded cable ribbon. In the robotic head presented in this paper, the real distance between the sensor and the controller is only 10 cm, as is shown in Figure 7a.
- Inertial sensor (IMU): This system consists of a 1,042 PhidgetSpatial precision 3/3/3 inertial sensor, which integrates a compass, a gyroscope and an accelerometer. Therefore, this sensor can perceive the movement and provide feedback of the information obtained by the control system of the motors. The position of the sensor on the proposed platform is shown in Figure 7b.
- Stereo audio system: The robotic head has an audio system based on two microphones and two speakers. This system uses a pre-amplifier M-AUDIO MOBILEPRE USB (preamp/audio interface) to control the audio inputs and outputs and, thus, reduce all the connections to a single cable.
- RGBD sensor: This device is composed of an RGB camera and a depth sensor (*i.e.*, an infrared projector combined with a monochrome CMOS sensor). Muecas integrates full compatibility with two different sensors: the Microsoft Kinect camera and Xtion PRO LIVE Sensor. These devices have features and computational power that make them useful for human-robot interaction, where the distance between the user and the robot is usually short and the priority is to estimate the user's pose.

### 3.3. Software Architecture

The multi-sensor robotic head, Muecas, uses the framework RoboComp [39] developed by the Robotics and Artificial Vision Lab (RoboLab) at the University of Extremadura. This software monitors and communicates the processes of the motors and sensors deployed in this platform via a computer and its connections to the hardware.

#### 3.3.1. RoboComp

**Figure 8.**
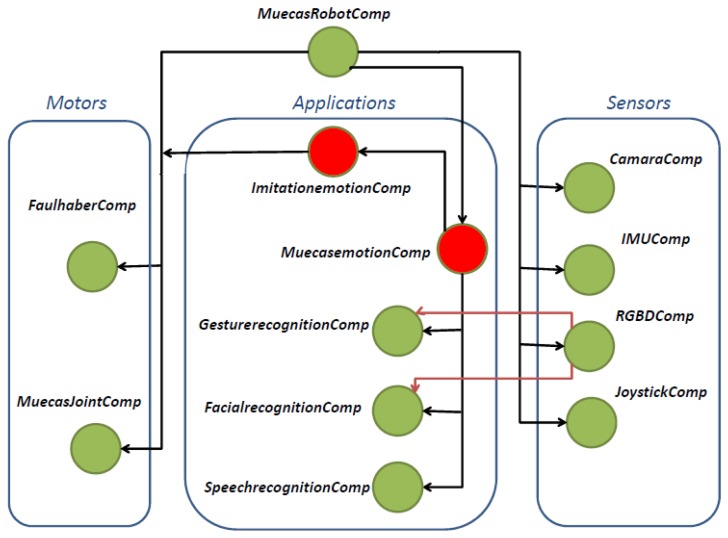
Dependence relationships between the different software components that control the hardware of the multi-sensor robotic head. All the components are programmed using the framework, RoboComp.

The software used to control the robotic head presented in this work is built on top of the robotics framework, RoboComp. Thanks to this framework, a homogeneous and efficient architecture capable of controlling the different subsystems of Muecas is developed. RoboComp in conjunction with the communication middleware, ICE (Internet Communications Engine middleware) [40], allow the use of software components, which can divide and communicate processes between different components. The software architecture and the dependence relationships are presented in Figure 8 and detailed in Table 2.

The main software component of the proposed system is MuecasRobotComp, which provides a control interface for all the elements of the robotic head, Muecas. The control of each motor depends on the mobile element and its movement mechanism. Therefore, the FaulhaberComp component controls the engines that communicate through the CAN-bus (*i.e.*, neck and eyes). The MuecasJointComp component communicates with the Pololu Micro Maestro-Servo Controller (*i.e.*, mouth and eyebrows). There are more software components associated with the different sensors of the platform.

#### 3.3.2. Speech System

The proposed design for the robotic head, Muecas, allows a broad compatibility with speech systems already implemented on Muecas: automatic speech recognition (ASR) and text to speech (TTS) systems. Specifically, Muecas is equipped with a TTS system that generates the mouth movement and the audio signal in a synchronous way [41]. Besides, Muecas also has an ASR system that recognizes the words to generate imitation and dialogue control systems.

## 4. Real-Time Emotion Recognition and Imitation Systems

**Figure 9.**
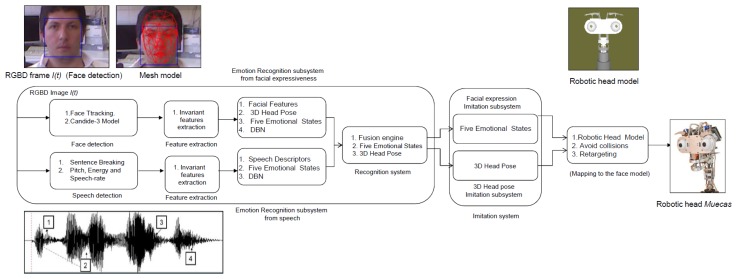
Overview of the imitation system proposed in this work.

In the affective human-robot interaction field, there are several studies currently being conducted to evaluate the benefits of the use of expressive robotic heads. Most of these studies are based on imitating different human behaviors and/or actions. In this work, a real-time approach that allows the robot to understand and imitate the emotional state of the user is developed. Besides, the robotic platform also imitates the movements of the head during the interaction. For these reasons, Muecas integrates a model-based representation of emotional states and pose, allowing it to imitate and respond to the emotions and actions of the user in real time. An overview of the proposed system is shown in Figure 9. A description of each module is given in the next subsections.

### 4.1. Emotional State Modeling

In recent years, the use of virtual models has been embraced for several robotic architectures. These new cognitive architectures build selective representations or models of the robot, the environment and the agents in it. Through these models, internal simulations that allow one to anticipate the outcome of future actions are performed (e.g., facial expressions, grasping of objects or more complex interactions). In this approach, the robotic head is represented by a virtual model, like an avatar, which is composed not only of the emotional state and the head pose estimate by the robot, but also of physical features and constraints (mesh model, limits of spin or the speed, among others). Therefore, Muecas' avatar is defined as *M*_{_*_robot_*_}_ = {(*m*_0_, *p*_0_), (*m*_1_, *p*_1_),…(*m*_5_, *p*_5_),*x, f, c*}, where *m_i_* represents an emotional state, *m_i_* ∈ {*happy, sad, anger, fear, neutral*}, for *i* = 1 to 5 and *p_i_*, the probability of this emotional state 0 ≤ *p_i_* ≤ 1 and ∑*_pi_* = 1, *x* is the 6-D vector of the head pose, *f* is the set of physical features of the robotic head (*i.e.*, mesh model) and *c* is defined as the set of physical constraints. *M_robot_* is updated once the facial expression and the emotional state of the user have been estimated.

### 4.2. Multimodal Emotion Recognition System

**Figure 10.**
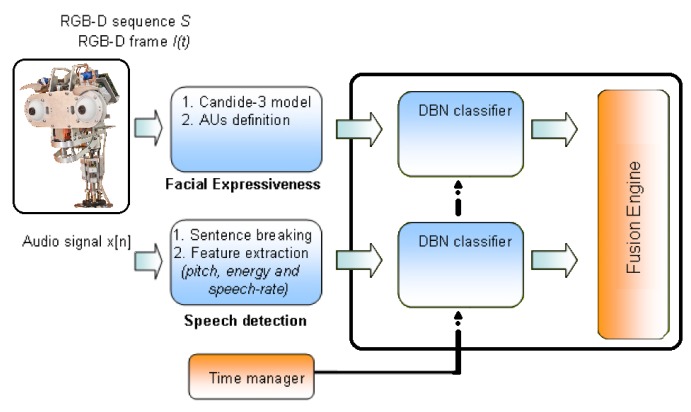
Overview of the multimodal emotion recognition system described in this work.

The multimodal emotion recognition system proposed in this paper is able to estimate the emotional state of the human based on different sources or modes. According to Ekman's work [19], five possible emotional states are estimated by the algorithm (*i.e.*, happy, sad, anger, fear and neutral). Facial expressiveness, in conjunction with speech, is analyzed in the proposal for emotion recognition. On the one hand, the facial expression recognition system obtains the user's emotional state through the analysis of the human face, evaluating the action units (AUs) from the Candide-3 reconstruction model [42] and using a dynamic Bayesian network (DBN) as the classifier. On the other hand, the robot uses the auditory information acquired in the conversation with the user to extract and analyze a set of independent descriptors. These descriptors are used in a second DBN to estimate the user's emotional state. Both systems run in parallel, and their results are fused in a third DBN. The multimodal recognition system proposed in this paper is illustrated in Figure 10. Each subsystem is briefly described below.

#### 4.2.1. Emotion Recognition System from Facial Expressiveness

**Figure 11.**
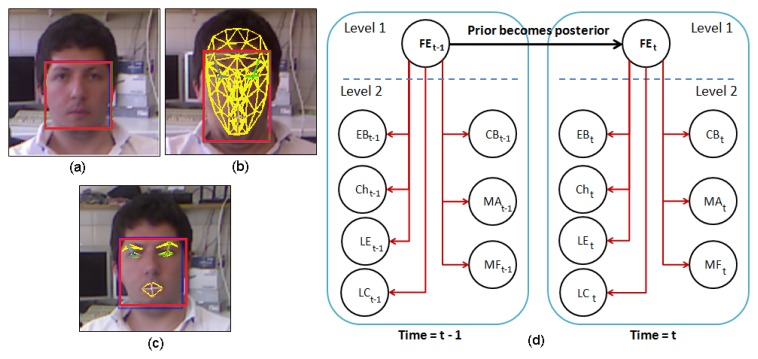
**(a)** RGB image of the user acquired by the sensor; (b) Candide-3 reconstruction model; (c) features extracted from the mesh model; and (d) dynamic Bayesian network; two time intervals are shown.

The proposed emotion recognition system for facial expressiveness consists of a robust feature extraction algorithm that uses the Candide-3 reconstruction model described in [42]. The proposal is inspired by the author's work [43], in which an RGB image is the input of the system. Contrary to this approach, an RGBD sensor is used in the presented work to extract a set of independent and antagonistic features of the user's face. An overview of the system described in this paper is shown in Figure 11. A description of each stage of the algorithm is given next.

- Data Acquisition: The facial expression recognition system proposed in this paper uses RGBD information acquired by the Microsoft Kinect sensor. This RGBD camera simultaneously provides two different images, a depth image and a color image (640 × 480) at real-time frame rates. The Kinect sensor is optimized to work from a close range of 0.5 m to 3 m, which allows one to use it for typical human-robot interaction.
- Face Tracking Algorithm: Let *S* be a sequence of RGBD images acquired by the robot in a real interaction, and let *I*(*t*) be the frame of this sequence at time *t*. Microsoft Kinect SDK, by means of its *FaceTracking* algorithm, is used for detecting and tracking the human face in real-time. The output of the algorithm is the list of points associated with the Candide-3 mesh model, *P^t^*. In Figure 11a, an RGBD image is acquired by the sensor. The mesh model is illustrated in Figure 11b.
- Invariant Facial Feature Extraction: The facial feature extraction algorithm proposed in this work is based on the analysis of the Candide-3 mesh model. The algorithm extracts a set of *m* relevant features from the retrieved Candide-3 mesh model, $F^{I}=\{f_{i}^{I}|i=1\ldots N\}$. The facial features used in this work are obtained directly from *P^t^*. These features, *f^I^*, are calculated using the Euclidean distances between different points in the mesh: (i) the upper contour of the eyebrows and the lower edge of the eyes, *d_eb_*; (ii) lip corners, *d_lc_*; (iii) upper contour and lower edge of the lips (mouth's aperture), *d_ma_*; and (iv) cheek, *d_ch_*. In order to become independent of the scale or distance from the user to the sensor, all these features are normalized by using the values extracted in the non-emotional state (*i.e.*, neutral). Figure 11c shows the set of features extracted by the algorithm, labeled as: *d_eb_* (yellow), *d_ch_* (yellow), *d_lc_* (white) and *d_ma_* (white).
- Dynamic Bayesian Network: The dynamic Bayesian network implemented in this system uses antagonistic properties in some AUs to increase the performance and to reduce the number of variables to be considered in the network. The proposed system uses only 11 action units, to reduce and optimize the information processing. These 11 AUs are grouped according to antagonistic and exclusive properties into only seven variables. These variables are the vectors *d_fe_* = {*EB, Ch, LE, LC, CB, MF, MA*} described in Table 3. The two-level network structure and the time influence that characterizes this classifier are illustrated in Figure 11d.

The first level of the DBN contains the belief variable, *FE*, representing the emotional state resulting from the classifier. Each facial expression is associated with a possible emotional state of the user, such as: *FE*_[_*_Neutral_*_]_, *FE*_[_*_Anger_*_]_, *FE*_[_*_Sad_*_]_, *FE*_[_*_Happy_*_]_ and *FE*_[_*_Fear_*_]_. The variables on the second level have as a parent this one on the first level: *FE*. The second level consists of the vector, *df_e_*.

The data, *D*, obtained in the facial feature extraction algorithm proposed in this paper, have the following set up:

(1) $$D=((x_{1},y_{1})\ldots (x_{n},y_{n})),x_{i}\in {\mathbb{R}}^{d},y_{i}\in \mathbb{R}$$

Consider that *y*_1_ to *y*_5_ are the five possible emotional states (*FE*_[_*_neutral_*_]_, *FE*_[_*_happy_*_]_, *FE*_[_*_sad_*_]_, *FE*_[_*_fear_*_]_, *FE*_[_*_anger_*_]_); and each dimension of *x* corresponds to one of the previously described random variables, namely: *EB, Ch, LE, LC, CB, MF* and *MA*. Since the learning data may have gaps between its samples, a model is built assuming that (*X*_1_, …, *X_n_*) are independent given *FE*, and:

(2) $$X_{i}\sim (\text{prio}r^{T}x_{i},\sigma^{2})$$

At first, *prior* ˜ *U*(1/*n*); however, throughout the iterations, the posterior of *t* − 1 becomes the prior on *t*.

Finally, by using Bayes's rule, we have the posterior equation:

(3) $$P(RE|x_{m})=\frac{\prod_{1}^{n}P(x_{i}|FE)*P(FE)}{P(x_{m})}$$

where *x_m_* is the most recent sensor data acquired.

The denominator can be computed using the Bayesian marginalization rule:

(4) $$P(x_{m})=\sum_{FE}\prod_{1}^{k}P(x_{i}|FE)*P(FE)$$

being *k* = 7, the number of random variables of the system.

In this Bayesian network, the model includes a dynamic convergence property over time, where the current frame uses the resulting histogram of the previous frame as prior knowledge. The process of convergence of this network limits the number of frames to five and the threshold to 80% to consider the complete classification. On the contrary, if the threshold is not exceeded after the fifth frame, the classifier selects the highest probability value (*i.e.*, the maximum *a posteriori* decision in Bayesian theory) as the correct classification result.

#### 4.2.2. Emotion Recognition System from Speech

**Figure 12.**
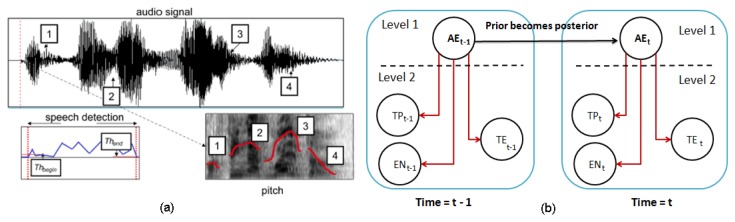
**(a)** The audio signal is processed in order to detect the beginning and ending of sentences, and then, a set of features is extracted (e.g., energy); (b) two time intervals for the dynamic Bayesian network classifier.

The proposed system for emotion recognition from speech uses a similar structure to the method described in Section 4.2.1. An audio descriptor defined as *d_s_* = {*Pt, En, Sr*} is used to estimate the human emotion, where *Pt, En* and *Sr* are pitch, energy and the speech rate of the audio signal, respectively. First, the audio signal, *x*[*n*], is pre-processed on-line in order to detect the presence or absence of speech (see Figure 12a). Then, the descriptor, *d_s_*, is calculated, as is shown in Table 4, considering *N* samples. The speech rate is calculated according to the following process: (1) the fast Fourier transform of the signal is performed; (2) the signal is multiplied with trains of pulses with different speech rates; and (3) the amount of energy at each rate is analyzed. Finally, the speech rate is calculated as the signal with the highest energy value. This descriptor is also the input of a DBN classifier, as is illustrated in Figure 12b.

#### 4.2.3. Multimodal Fusion for Emotion Recognition

**Figure 13.**
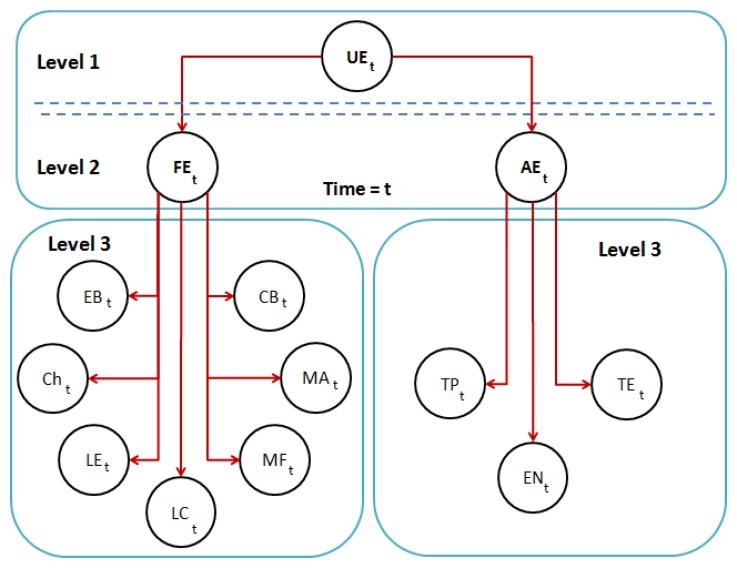
The last dynamic Bayesian network (DBN) classifier combines the estimate of each subsystem.

The proposed fusion system analyzes the information obtained by the emotion recognition systems based on facial expressions and speech, and thus, the number of errors in the human emotion estimation is reduced. The decisions of each emotion recognition subsystem are combined to a fused decision vector that is analyzed by the Bayesian network of three levels (see Figure 13) in real time. As shown in the figure, the node, *U_E_*, is the parent of the nodes of the second level, *FE* and *AE*. For each node on the first and second level (*i.e., U_E_*, *FE* and *AE*), there are only five possible results (neutral, happiness, fear, anger, sadness). The time manager in Figure 10 directly synchronizes the output of all the classifiers.

### 4.3. Imitation System for HRI

**Figure 14.**
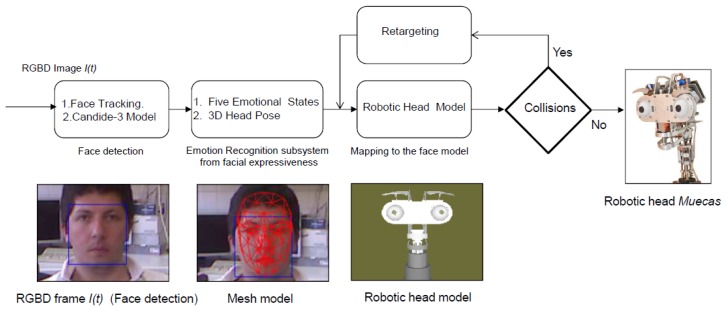
Overview of the imitation system for human-robot interaction (HRI) proposed in this paper.

In this section, the system for real-time imitation proposed in this paper is presented. The proposal consists of two systems running in parallel to imitate both the facial expressions and the body language of the user's head. Once the emotion has been estimated by the multimodal emotion recognition module, the system updates the virtual model of the multi-sensor robotic head, *M*_{_*_robot_*_}_. Next, the system imitates the movements and facial expressions through the set of motors and mobile elements of Muecas. On the one hand, the facial expression imitation system is capable of imitating the same facial expressions that are recognized by the emotion recognition system. Therefore, this system is also based on action units. On the other hand, the head's pose imitation system is able to imitate the basic movements of the head. An overview of the proposed imitation system is illustrated in Figure 14, and each subsystem is briefly described below.

#### 4.3.1. Facial Expressions Imitation System

**Figure 15.**
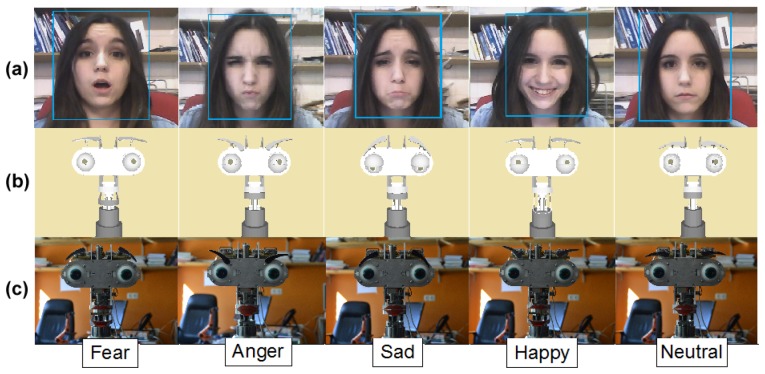
**(a)** Facial expression of the user; (b) model-based representation of each emotional state; and (c) imitation of each facial expression by the robotic head, Muecas.

Similar to the facial expression recognition system, the imitation of facial expression in the proposed work is based on the Facial Acting Coding System. Table 5 shows the mobile elements of the robotic head and the action units associated with each emotional state. Thus, once the virtual model of the robotic head is updated, *M*_{_*_robot_*_}_, the kinematic chain associated with the emotional state is evaluated. In case of collisions due to the physical constraints of the robotic head, a retargeting of each mobile element is performed to generate a new kinematic chain over the virtual model. Figure 15a illustrates five different human facial expressions recognized by the system. The model-based representation for each facial expression is illustrated in Figure 15b. Finally, the imitation through the robotic head is shown in Figure 15c.

#### 4.3.2. 3D Head Pose Imitation System

**Figure 16.**
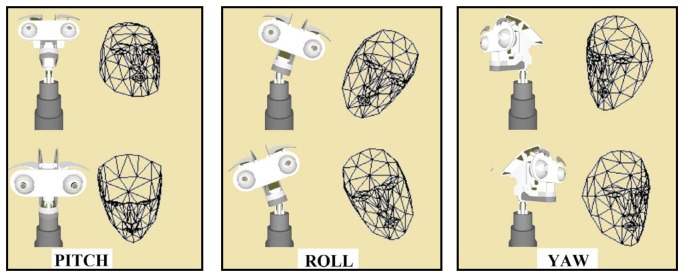
Degrees of freedom of the robotic neck in the imitation stage.

The Candide-3 face model is used in this work to imitate the body language of the user's head. The model-based representation is used not only to imitate the movements of the user, but also to keep the ocular globes of the robot focused on the human face. Figure 16 shows the different degrees of freedom of the robotic neck (*i.e.*, pitch, roll and yaw) that are able to be imitated by the system. In Table 6, the action units associated with each movement is described. The process of imitation is similar to the one previously mentioned for the imitation of facial expressions, starting with the information about the position and orientation of the user obtained through the mesh model. Then, this information is used to generate the mapping on the virtual representation of this platform and to get the kinematic chain for each movement, avoiding collisions and generating a natural movement for the human observer.

## 5. Experiments Results

**Figure 17.**
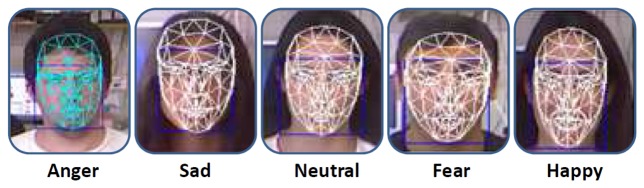
Sample of users in the tests described in this work.

In this section, a set of tests has been carried out in order to evaluate the emotion recognition and imitation systems of the multi-sensor robotic head, Muecas. On the one hand, the recognition and imitation of facial expressions is evaluated. A set of tests evaluate the performance of the facial expression recognition system through the Candide-3 reconstruction model. In addition, these tests evaluate the functionality and naturalness of the facial expressions generated on the presented platform. On the other hand, new tests are conducted to verify the functionality and efficiency of the design of the robotic head, as well as the robustness of the system of imitation of head movements. The same group of users, with different characteristics, genders and ages, has been used for both experimental tests. Figure 17 shows a sample of users, where the Candide-3 mesh model and the emotion estimate by the system have been drawn.

### 5.1. Facial Expression Imitation by the Robotic Head

**Figure 18.**
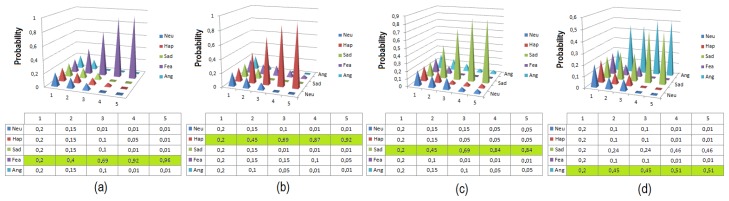
Results from the DBN, in the tests for imitation based on facial expression; (**a**) fear; (**b**) happy; (**c**) sad; and (**d**) anger.

The first tests are conducted using a group of 40 people with different genders and facial features. The tests consist of five random sequences of facial expressions performed by each user, as is described in previous work by the same authors [42]. The sequence must contain each facial expression at least once in a random order. The robustness and homogeneity of the results of this system are then evaluated. To consider a test successful, the system must recognize and imitate perfectly each facial expression through the multi-sensor robotic head. In Table 7, the percentage of facial expressions correctly detected and imitated, *P_fe_*, is shown. All facial expressions are detected with a percentage of success always higher than 90%, which demonstrates the correct operation of the emotion recognition and imitation system. Figure 18 illustrates the results of the dynamic Bayesian network of the facial expression recognition system. As shown in the figure, after five times, the emotion is recognized by the method with high probability

The results obtained from the facial expressions recognition can be compared with those obtained by similar works (Table 8). In previous works by the same authors [43], a sequence of RGB images was used for recognizing a set of basic emotions from facial expressions using Gabor filtering (a method that had several limitations based on the distance to the sensor). Others similar works are evaluated, which use the Cohn-Kanade Facial Expression Database and the mesh model Candide-3, but with different classification systems: Bayesian network [11] and model tree [10]. In order to perform a comparative study, four different aspects are taken into account: accuracy, the number of human emotions recognized by the algorithms, methods and classification systems.

### 5.2. 3D Head Pose Imitation System

The evaluation of the 3D head pose imitation system is based on previous work [7]. In that work, the position and orientation of the user's head was evaluated using an RGB sensor. In this paper, the algorithm uses RGBD information and the Candide-3 mesh model. The tests consist of the recognition and imitation of specific movements of the user's head. Similar to the evaluation of the facial expression recognition and imitation system, the users perform a sequence of pitch, roll and yaw movements in front of the robot (see Figure 16). Each movement is repeated 150 times with different lighting conditions and a different speed of movement. Table 9 shows the percentages of correctly estimated poses, *P_m_*. As shown in the table, the roll and yaw movements present the best results, contrary to the pitch movement that has a *P_m_* equal to 93 percent.

### 5.3. Emotion Recognition from Speech

The third test checks the robustness of the emotion recognition from speech. It analyzes the sentences in the audio signal to estimate the emotional states of the user. For this test, the same group of people is used. In Table 10, the results show the percentages of the emotional states correctly detected, *P_AE_*. In this method, the emotions with less intensity showed better results in the recognition from speech.

Finally, the details of the errors of the method described in Sections 5.1 and 5.3 are illustrated in Table 11.

## 6. Conclusions and Future Work

Current social robotics require the development of new agents for interacting in certain social environments, such as robotic care (care for the elderly and people with disabilities), rehabilitation and education, among others. This interaction is usually based on verbal and nonverbal communication. Therefore, the multi-sensor robotic head, Muecas, for affective human-robot interaction is presented in this paper. Muecas is able to be integrated with different robotic platforms, while maintaining its ability to convey emotional information. The design of the robotic head has followed the main psychological theories about the human acceptance of robots, and thus, Muecas is based on an anthropomorphic and caricatured design. This platform is composed of a set of sensors and actuators that allows affective interaction with humans based on natural language. On the one hand, the robot is equipped with RGB and RGBD sensors to acquire color and depth information from the environment. Besides, the robot has an inertial sensor and an audio system composed of two microphones and two speakers. On the other hand, the mechanical design of the robot includes a set of mobile elements and motors that allow the robotic platform to perform several affective movements identifiable by an untrained user (*i.e.*, to generate facial expressions or to imitate human body language according to pitch, yaw and roll movements). The hardware and software architectures have been described in this paper.

Regarding the emotion recognition system, the proposal uses an original multimodal approach, where also the emotion recognition from speech is an original contribution of this paper. This multimodal emotion recognition system has been compared with previous works, demonstrating the improvement of the results using different information channels. On the one hand, the system implements human emotion recognition from facial expressions, which uses the Candide-3 mesh model and a dynamic Bayesian network classifier. On the other hand, human emotion recognition from speech is also presented. This Bayesian approach to the emotion recognition problem presents good results for real-time applications with untrained users in an uncontrolled environment. Once the emotion has been estimated, the system of imitation uses the information to update the internal model of the robotic platform. This representation is constructed through a virtual model that generates the kinematic chain for every facial expression and movement, which avoids collisions between the mobile elements and allows one to regenerate the kinematic chain before generating the real movements. The implementation of this representation is performed using a cognitive architecture that allows one to update the emotional state and the movement of the models, giving rise to a system that controls the process of interaction based on the body language skills of the robot.

Future work will focus on new methods of affective interaction to improve the level of empathy and the user's attention to the robot. The movements of the eyelids, as well as the use of a cover for the robot face should be evaluated. Besides, it is also interesting that the robot is able to learn how to respond to different human emotions. Therefore, an interesting topic that directly uses the results of this paper is to implement an emotion learning system for the multi-sensor robotic head, Muecas.

## Figures and Tables

**Table 1.** Comparison of different robotic heads in the literature (Ant–anthropomorphic; Tech–technomorphic; Zoo–Zoomorphic). FACS, Facial Action Coding System.

**Robotic Head**	**WE-4RII**	**ROMAN**	**SAYA**	**KHH**	**BARTHOC**	**iCub**	**iCat**
Neck DoF	4	4	3	4	4	3	2
Eyebrow DoF	8	2	no	no	2	LED	2
Eye DoF	3	2	2	3	3	3	0
Mouth DOF	5	1	1	no	3	LED	5
FACS	yes	yes	yes	no	yes	no	no
Stereo Vision	yes	yes	no	yes	yes	yes	no
Stereo Audio	yes	no	yes	yes	no	yes	yes
RGBD Sensor	no	no	no	no	no	no	no
Inertial Sensor	yes	yes	yes	yes	no	yes	no
Appearance	Ant.	Ant.	Ant.	Tech.	Ant.	Ant.	Zoo.

**Table 2.** Software components, sensors and actuators of the multi-sensor robotic head.

**Components**	**Sensor or Actuator**	**Description**
FaulhaberComp	Faulhaber (LM-1247 - LM-2070)	Control of the engines of the eyes
FaulhaberComp	Faulhaber (1724-024 SR)	Control of the engines of the neck
MuecasJointComp	HITEC HS-45HB	Control of the engines of the eyebrows and mouth
CamaraComp	Point Grey Dragonfly2 IEEE-1394	Control of the stereo vision system
IMUComp	PhidgetSpatial 1042_0 - 3/3/3	Control of the inertial system
RGBDComp	Microsoft Kinect Sensor	Acquires the data from the RGBD sensor
JoystickComp	Logitech Attack 3	Acquires the data of the joystick

**Table 3.** Facial expressiveness vector *d_fe_*.

**Variable**	**Element of the Face**	**Action Units (AUs)**
*EB*	Eyebrows	{*AU*1, *AU*4, *none*}
*Ch*	Cheeks	{*AU*6, *none*}
*LE*	Lower Eyelids	{*AU*7, *none*}
*LC*	Lip Corners	{*AU*12, *AU*15, *none*}
*CB*	Chin Boss	{*AU*17, *none*}
*MF*	Mouth's Form	{*AU*20, *AU*23, *none*}
*MA*	Mouth's Aperture	{*AU*24, *AU*25, *none*}

**Table 4.** Speech features vector *d_s_. P_t_*, the pitch range is calculated using the HPS (harmonic product spectrum) algorithm [44].

*Pt*	Pitch	$\max\{Y(\omega )=\prod_{r=1}^{i=R}{\left|X(\omega r)\right|}^{2}\}$
*En*	Energy	$E=\frac{1}{N}\cdot \sum_{x=0}^{x=i}x{\left[i\right]}^{2}$

**Table 5.** Movements of the robotic head, Muecas, and mobile elements associated with each emotional state.

**Emotion**	**AUs**	**Mobile Components of Muecas**
Neutral	-	-
Happy	AU6-AU12-AU25	Eyebrows-Eyelids-Eyes-Mouth
Sad	AU1-AU4-AU15-AU17	Eyebrows-Eyelids-Eyes
Fear	AU1-AU4-AU20-AU25	Eyebrows-Eyelids-Mouth
Anger	AU4-AU7-AU17-AU23-AU24	Eyebrows-Eyelids

**Table 6.** Movements of the robotic head, Muecas, and the action units associated with the face tracking algorithm.

**Head Movements**	**AUs**	**Muecas Movements**
Yaw	AU51-AU52	Head turn left-Head turn right
Pitch	AU53-AU54	Head up-Head down
Roll	AU55-AU56	Head tilt left-Head tilt right
Eye pan	AU61-AU62	Eyes turn left-Eyes turn right
Eye tilt	AU63-AU64	Eye up-Eye down

**Table 7.** Robustness of the system of recognition and imitation of facial expressions.

**Test** *P_fc_*	**Sadness**	**Happiness**	**Fear**	**Anger**	**Neutral**	**Errors**
Sadness	**90%**	0%	0%	0%	4%	6%
Happiness	0%	**98%**	0%	0%	0%	2%
Fear	1%	2%	**95%**	0%	0%	2%
Anger	2%	0%	0%	**95%**	0%	3%
Neutral	3%	0%	0%	0%	**92%**	5%

**Table 8.** Comparison between different emotion recognition methods.

	Proposed Method	Previous Work [43]	Riaz *et al.* [11]	Mayer *et al.* [10]
**Accuracy**	94%	93%	90%	87%
**No. of Emotions**	5	5	6	6
**Method**	Candide-3	Gabor	Candide-3	Candide-3
**Classifier**	Bayesian network	Bayesian network	Bayesian network	Model tree

**Table 9.** Robustness of the pose imitation.

**Test**	**Percentage of Correctly Estimated Poses** *P_m_*
Pitch	93%
Roll	98%
Yaw	95%

**Table 10.** Robustness of the emotion recognition from speech.

**Test** *P_AE_*	**Sadness**	**Happiness**	**Fear**	**Anger**	**Neutral**	**Errors**
Sadness	**87%**	0%	0%	0%	2%	11%
Happiness	0%	**71%**	5%	0%	0%	24%
Fear	2%	2%	**67%**	5%	0%	24%
Anger	2%	0%	5%	**78%**	0%	15%
Neutral	5%	0%	0%	0%	**82%**	13%

**Table 11.** Details of the errors, *P_fe_* and *P_AE_*.

**Errors**	**Misclassification**	**Ambiguous**	**Under Threshold**
*P_Test_*_1_(*FE*)	2%	1%	1%
*P_Test_*_3_(*AE*)	11%	2%	5%
